# Sublethal Toxicity Endpoints of Heavy Metals to the Nematode *Caenorhabditis elegans*

**DOI:** 10.1371/journal.pone.0148014

**Published:** 2016-01-29

**Authors:** Ying Jiang, Jiandong Chen, Yue Wu, Qiang Wang, Huixin Li

**Affiliations:** 1 College of Resources and Environment, Henan Agricultural University, Zhengzhou, 450000, P.R. China; 2 College of Resources and Environmental Sciences, Nanjing Agricultural University, Nanjing, 210095, P.R. China; 3 School of Management Science and Engineering, Guangxi University of Finance and Economics, Nanning, 530003, P.R. China; 4 Soil and Fertilizer Bureau of Shandong Province, Jinan, 250100, P.R. China; Chinese Academy of Sciences, CHINA

## Abstract

*Caenorhabditis elegans*, a free-living nematode, is commonly used as a model organism in ecotoxicological studies. The current literatures have provided useful insight into the relative sensitivity of several endpoints, but few direct comparisons of multiple endpoints under a common set of experimental conditions. The objective of this study was to determine appropriate sublethal endpoints to develop an ecotoxicity screening and monitoring system. *C*. *elegans* was applied to explore the sublethal toxicity of four heavy metals (copper, zinc, cadmium and chromium). Two physiological endpoints (growth and reproduction), three behavioral endpoints (head thrash frequency, body bend frequency and feeding) and two enzymatic endpoints (acetylcholine esterase [AChE] and superoxide dismutase [SOD]) were selected for the assessment of heavy metal toxicity. The squared correlation coefficients (R^2^) between the responses observed and fitted by Logit function were higher than 0.90 and the RMSE were lower than 0.10, indicating a good significance statistically. There was no significant difference among the half effect concentration (EC50) endpoints in physiological and behavioral effects of the four heavy metals, indicating similar sensitivity of physiological and behavioral effects. AChE enzyme was more sensitive to copper, zinc, and cadmium than to other physiological and behavioral effects, and SOD enzyme was most sensitive to chromium. The EC50 of copper, zinc, and cadmium, to the AChE enzyme in the nematodes were 0.68 mg/L, 2.76 mg/L, and 0.92 mg/L respectively and the EC50 of chromium to the SOD enzyme in the nematode was 1.58 mg/L. The results of this study showed that there was a good concentration-response relationship between all four heavy metals and the sublethal toxicity effects to *C*. *elegans*. Considering these sublethal endpoints in terms of simplicity, accuracy, repeatability and costs of the experiments, feeding is the relatively ideal sublethal toxicity endpoint of heavy metals to *C*. *elegans*.

## Introduction

Heavy metal pollution is of serious concern, along with the development of human production activities, industrial and agricultural waste water emissions. Heavy metals are persistent environmental pollutants and harmful to health and the environment. They often appear in sublethal doses of animals and plants on the aquatic environment, which indicates that exposure to low concentration characteristics [1, 2]. Therefore, to study the sublethal effects of these agents, and to monitor their risk for humans and the environment is very important.

*Caenorhabditis elegans* is a widespread, free-living nematode that lives mainly in the interstitial pore water of soil. With a short life cycle (3–4 days at 20°C), small size (1 mm), and transparency characteristics, *C*. *elegans* is easy breeding with low cost of culturing. Synchronized cultures can be obtained from either eggs or daubers. Its genome is fully sequenced and available on public database. This makes *C*. *elegans* an excellent model in various toxicological studies. Using *C*. *elegans* as a model organism, a fairly systematic evaluation system of sublethal concentration endpoints has been established after years of researches. The endpoints mainly include: lifespan [3–5], cell apoptosis [6,7], growth and development [5,8], reproduction [9,10], motion behavior and feeding behavior [11,12], enzymes such as AChE and SOD [13,14], and molecular and genetic endpoints including heat shock proteins and relevant genetic expression [15–17].

The choice of an optimal endpoint for testing toxicants may depend on several factors, including sensitivity, accuracy, and maneuverability. Sensitive endpoints permit detection of lower levels of toxicants, may allow the use of shorter testing intervals, or may provide insight into the mechanism of action of toxicants. The current *C*. *elegans* literature provides a certain degree of insight into the relative sensitivity of several endpoints; however, few experiments provide direct comparisons of multiple endpoints under a common set of experimental conditions. Behavior and reproduction responses of *C*. *elegans* were compared and found to be similar and both are much more sensitive indicators of toxicity of ethanol than lethality [18], but its sensitivity relative to other sublethal endpoints remains uncertain. The possible correlation between the two sublethal endpoints of toxicity, feeding and acetylcholin esterase (AChE) activity in transgenic *C*. *elegans* (hsp16-lacZ) exposed to sublethal concentrations of dichlorvos was examined [19]. Four sublethal endpoints, life span, development, reproduction, locomotion behavior and chemotaxis plasticity, were employed to discover the toxicity of Zn exposed to progeny of *C*. *elegans* [9]. The sensitivity of growth, reproduction and stress-related gene expression was compared for exposure to di(2-ethylhexyl)phthalate (DEHP) [15]. Therefore, it is necessary to test multiple endpoints of *C*. *elegans* under a common set of experimental conditions.

The objectives of this study were to compare growth, reproduction, head thrash frequency, body bend frequency, feeding, AChE and SOD as sublethal endpoints for heavy metal toxicity tests, using *C*. *elegans* as a model organism, and to identify a suitable tool to develop a screening system for ecotoxicity monitoring. To this end, four heavy metals (copper, zinc, cadmium and chromium) were tested, in expectation of determining a speedy, accurate and cost-efficient endpoint for the sublethal toxicity of heavy metals and providing time-efficient and accurate methodological basis and technical support for the evaluative studies of the ecological risks of heavy metals.

## Materials and Methods

No specific permissions were required for the described field studies. We confirmed that the field studies did not involve endangered or protected species.

### 2.1 Test Organisms

*Caenorhabditis elegans* wild type strain N2, was originally obtained from the *Caenorhabditis* Genetics Center (CGC) (Minnesota, USA). The worms were maintained on nematode growth medium (NGM) plates seeded with OP50 (a uracil-deficient strain of *Escherichia coli*) at 20°C as described by Brenner [20]. A stock solution of dauerlarvae in M9 buffer was prepared, kept at 20°C, and renewed monthly [21]. The dauers were used to generate age synchronous adult worms for toxicity tests. The dauers were placed on NGM plates with a lawn of OP50 for a food source and were incubated at 20°C. After 3 days, gravid nematodes were washed off the plates into centrifuge tubes and were lysed with a bleaching mixture (0.45 mol/L NaOH, 2%HOCl), which killed all life stages except for eggs [22]. The eggs were collected and placed on NGM plates and incubated to obtain age-synchronous young adults. Three day old nematodes were collected and rinsed with K-medium (0.032M KCl, 0.051M NaCl) at least three times [23].

### 2.2 Test chemicals and analysis

Four analytical grade chemicals were used: copper sulfate pentahydrate (CuSO_4_·5H_2_O, Camycal, 98%), zinc chloride (ZnCl_2_, Sigma-Aldrich, 98%), cadmium chloride (CdCl_2_, Sigma-Aldrich, 98%) and potassium dichromate (K_2_Cr_2_O_7_, Camycal, 98%).

All metal solutions were prepared by dissolving their salts in K-medium. The concentration of all metal solutions was analyzed by inductively coupled plasma spectrometer (ICP). The results showed that measured concentrations varied generally less than 5% from the nominal concentrations. Thus, all calculations were based on nominal concentrations.

### 2.3 Test Design

The four heavy metals (copper, zinc, cadmium, chromium) were dissolved in K-medium respectively. According to the results of preparatory experiment, each heavy metal was diluted at the ratio of 0.5 and was prepared in five successive concentration gradients in order to evenly cover the inhibitory effect range from 10% to 90%. The five concentrations (mg/L) of the four heavy metals were as follows:

Cu: 10, 5, 2.50, 1.25, 0.63;

Zn: 50, 25, 12.50, 6.25, 3.13;

Cd: 20, 10, 5, 2.50, 1.25;

Cr: 100, 50, 25, 12.50, 6.25.

K-medium without any heavy metal was used as controls. The following five physiological and behavioral endpoints were determined: growth, reproduction, head thrash frequency, body bend frequency, and feeding.

#### 2.3.1 Growth

Age-synchronous adult Nematodes (10±1) and test solution (2ml) were added to each well of a 12-well cell culture plate, with four wells for each concentration and twelve wells for the controls. The experiment was repeated three times. *E*. *coli* OP50 (10 μl) was added to each well as food source to prevent nematodes possible death or other effects due to starvation. The 12-well plate was placed in incubator and incubated at 20°C for 24 hours. The body lengths of the nematodes after exposure to toxicants were measured respectively with microscope and the Image-Pro Express Software. The number of measured nematodes for the exposed group and the control group was 15 (picked at random from repetitions of each concentration gradient and the controls). The growth inhibition (%) = (1—body length change of exposed nematode at each concentration / body length change of control nematode) × 100.

#### 2.3.2 Reproduction

Age-synchronous adult Nematodes (1 worm) and test solution (2 ml) were added to each well of a 12-well cell culture plate, with six wells for each concentration and twenty wells for the controls. The experiment was repeated three times. *E*. *coli* OP50 (10 μl) was added to each well as food source to prevent nematodes possible death or other effects due to starvation. The 12-well plate was placed in incubator and incubated at 20°C for 72 hours. Production rates were determined based on the method described by Middendorf and Dusenbery (1993) [24]. The number of progenies, i.e. larvae at various instars excluding eggs, of each nematode at the end of 72 hours of exposure was recorded. The reproduction inhibition (%) = (1—reproduction rate of exposed nematodes / reproduction rate of control nematodes) × 100.

#### 2.3.3 Head Thrash Frequency and Body Bend Frequency

Head thrash frequency was measured by following the method described by Tsalik et al (2003) [25]. Briefly, M9 buffer (30 μl) was added in drops to a new NGM plate. The nematodes were placed in the solution after 24 hours of exposure to heavy metals. After 1 min recovery period, the numbers of their head thrashes in 1 min were recorded. One head thrash was counted when the body bend of the nematode reached half of its body length. The nematodes that stopped head thrash for over five seconds were not counted. Fifteen nematodes were measured for the exposed group and the control group respectively (picked at random from repetitions of each concentration gradient and the controls).

Body bend frequency was determined based on the methods described by Tsalik et al. (2003) [25]. Nematodes were placed in an NGM plate without OP50 after 24 hours of exposure and the numbers of their body bends in 20 seconds were recorded. Assuming the movement along the pharyngeal pump was the y axis, each body bend was defined as one change of the body in direction of the corresponding x axis. The number of measured nematodes for the exposed group and the control group was 15 respectively (picked at random from repetitions of each concentration gradient and the controls).

The temperature of the laboratory was set at 20°C and a microscope with cold light source was used to exclude any possible effect of temperature fluctuation on the behavior of nematodes. The head thrash inhibition (%) = (1—head thrash frequency of exposed nematodes / head thrash frequency of control nematodes) × 100. The body bend inhibition (%) = (1—body bend frequency of exposed nematodes / body bend frequency of control nematodes) × 100.

#### 2.3.4 Feeding

Feeding was performed based on methods described by Jones et al. [26], with some modifications. The *E*. *coli* OP50 was resuspended and diluted, with K-medium or K-medium containing heavy metals, to OD≈ 1.0 (590 nm). Age-synchronous adult nematodes (2000±100) and test solution containing *E*. *coli* OP50 (2 ml) were added to each well of a 12-well cell culture plate, with four wells for each concentration and twelve wells for the controls. The 12-well plates were placed in a constant temperature shaking table (120 rpm) and incubated at 20°C for 24 hours. Before determination of the OD values, the 12-well plate stayed standing for 15 minutes such that the nematodes in the solution were precipitated, then 300 μl of supernatant was extracted from each well and placed in a 96-well cell culture plate. The OD values (590 nm) were read using Microplate Reader (E11383, Molecular Devices, USA). Two controls were set in the experiment (K-medium + OP50, and K-medium + OP50 + heavy metals) in order to calibrate the death or proliferation of *E*. *coli* OP50 cells due to the heavy metals toxicity. The experiment was repeated for three times.

The expression of the feeding inhibition of the nematodes *E*(%):

$$E\;(\%)=\left(1\;-\;\frac{{\Delta \text{OD}}_{\text{heavy metal}(+\text{worm})}-{\Delta \text{OD}}_{\text{heavy metal}(-\text{worm})}}{{\Delta \text{OD}}_{\text{K}-\text{medium}(+\text{worm})}-{\Delta \text{OD}}_{\text{K}-\text{medium}(-\text{worm})}}\right)\times 100$$ (1)

in which ⊿OD stands for the difference in OD values before and after exposure to toxicants.

#### 2.3.5 Acetylcholin esterase and Superoxide dismutase

The four heavy metals (copper, zinc, cadmium, chromium) were respectively dissolved in K-medium. According to results of preparatory experiment, each heavy metal was prepared in five successive concentration gradients in order to evenly cover the inhibitory effect range from 10% to 90%. The five concentrations (mg/L) of the four heavy metals were as follows:

Cu: 10, 5, 1, 0.10, 0.01;

Zn: 30, 20, 10, 1, 0.10;

Cd: 50, 30, 10, 1, 0.10;

Cr: 20, 10, 5, 1, 0.10.

The enzymatic activities of acetylcholin esterase (AChE) and superoxide dismutase (SOD), were determined using AChE kit and SOD kit (Nanjing Jiancheng Bioengineering Institute), respectively. The enzymatic activity inhibition (%) = (1—enzymatic activity of exposed nematodes / enzymatic activity of control nematodes) × 100.

### 2.4 Concentration–Response Curve Fitting

The concentration-response relationship was expressed with the concentration-response curve (CRC), The X-axis plots concentration of heavy metals and the Y-axis plots response. Ordinarily, the modeling of the abscissa-response relationship is based on the assumed absence of randomness or error, so that the response (y) may be calculated from the concentration (x) through fixed functional relationship. The optimum regression model was selected based on the squared correlation coefficients (R^2^) and the root mean square errors (RMSE) between the observed values and the fitted values. The greater the R^2^ value (closer to 1) and the less the RMSE value (closer to 0), the better the prediction ability of the model is.

After selection of the models, the concentration-response relationship of the four heavy metals in the experiment was mainly described using the two-parameter Logit model and Weibull model. Both the Logit and Weibull models were initially applied in quantum data and were distributed based on simple cumulative statistics. The assumed CRC was reflectionally symmetrical in the Logit function with the median effective concentration line (E = 50%) as the line of symmetry, while the Weibull function was asymmetrical, thereby applicable in more curves. Through the non-linear fitting of the concentration-response statistics of the heavy metals and their mixtures, the simulated CRC parameters *α* and *β* were acquired, thereby determining the corresponding fitting functions and calculating the value of ECx, i.e. the effect concentration of the heavy metals to the nematode.

The corresponding functions of the Logit model and the Weibull model were as follows:

Logit:

E=1/(1+exp(−α−β*log10(c)))

Weibull:

E=1−exp(−exp(α+β*log10(c)))

where E stands for effect, *α* and *β* stand for model parameters, and *c* stands for concentration.

### 2.5 Processing of Data and Diagramming

The data statistics and significance test were conducted using SPSS18.0 (Statistical Product and Service Solutions); the non-linear data fitting and calculation were conducted using 1stOpt (First Optimization); the diagramming were conducted on Origin8.5. The mean values of three experiments were used in data for the fitting and statistics.

## Results and Analysis

Considering that the difference between theoretical content and measured content of the heavy metals was less than 5%, all calculations of concentrations for this experiment were based on theoretical concentrations. After the non-linear fitting of the concentration-response data, the R^2^ between the responses observed and fitted by Logit function are higher than 0.90 and the RMSE are lower than 0.10, whereas the R^2^ between the responses observed and fitted by Weibull function are higher than 0.88 and the RMSE are lower than 0.16. Consequently, the Logit model was superior to the Weibull model in fitting degree. Therefore, the calculation of EC50 was based on fitting in the Logit model. The EC50 of the four heavy metals to *C*. *elegans* for each physiological, behavioral and enzymatic endpoint and their 95% confidence intervals are shown in Table 1.

**Table 1:** EC50 of four heavy metals exposures for each of seven sublethal endpoints and their 95% confidence intervals.

Endpoint	EC50 (mg/L)			
	Cu	Zn	Cd	Cr
**Body length**	3.2ab[2.84–3.63]	15.13a[9.59–24.15]	5.74b[4.12–8.04]	15.32a[10.58–21.1]
**Head thrash frequency**	3.57ab[2.37–5.51]	12.42ab[10.23–15]	8.41b[6.68–10.93]	19.07a[12.89–26.08]
**Body bend frequency**	4.24a[3.91–4.57]	15.92a[11.54–22.67]	8.04b[4.49–16.28]	18.63a[13.68–34.43]
**Feeding**	3.32ab[2.08–5.13]	12.6ab[10.43–15.1]	5.2b[4.37–6.23]	18.45a[14.16–23.51]
**Reproduction**	3.31ab[2.09–5.61]	14.12ab[11.1–18.07]	5.88b[4.79–7.23]	18.39a[13.91–23.77]
**Superoxide dismutase (SOD)**	2.81b[1.93–3.79]	11.45b[6.12–15.92]	15.05a[11.49–19.67]	1.58b[0.82–2.48]
**Acetylcholine esterase (AChE)**	0.68c[0.34–1.24]	2.76c[1.89–3.95]	0.92c[0.58–1.40]	11.91a[9.66–15.29]

Note: Data in square brackets show the 95% confidence intervals; Different letters in the same column indicate significant differences (*p* < 0.05) among endpoints.

### 3.1 Physiological and Behavioral Effects of Heavy Metals to *C*. *elegans*

**Fig 1:**
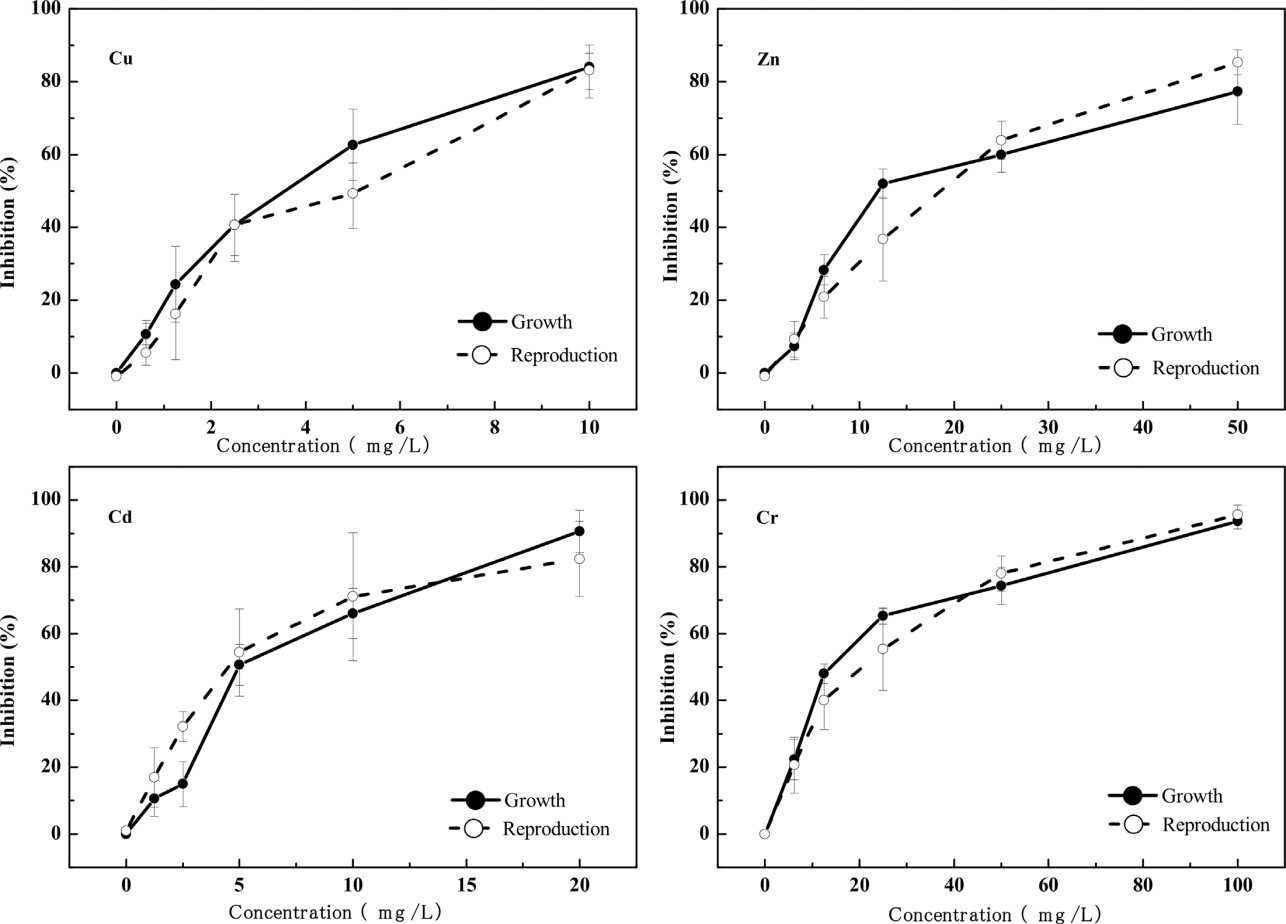
Concentration-response relationships for Cu, Zn, Cd, Cr exposures for body length and reproduction.

**Fig 2:**
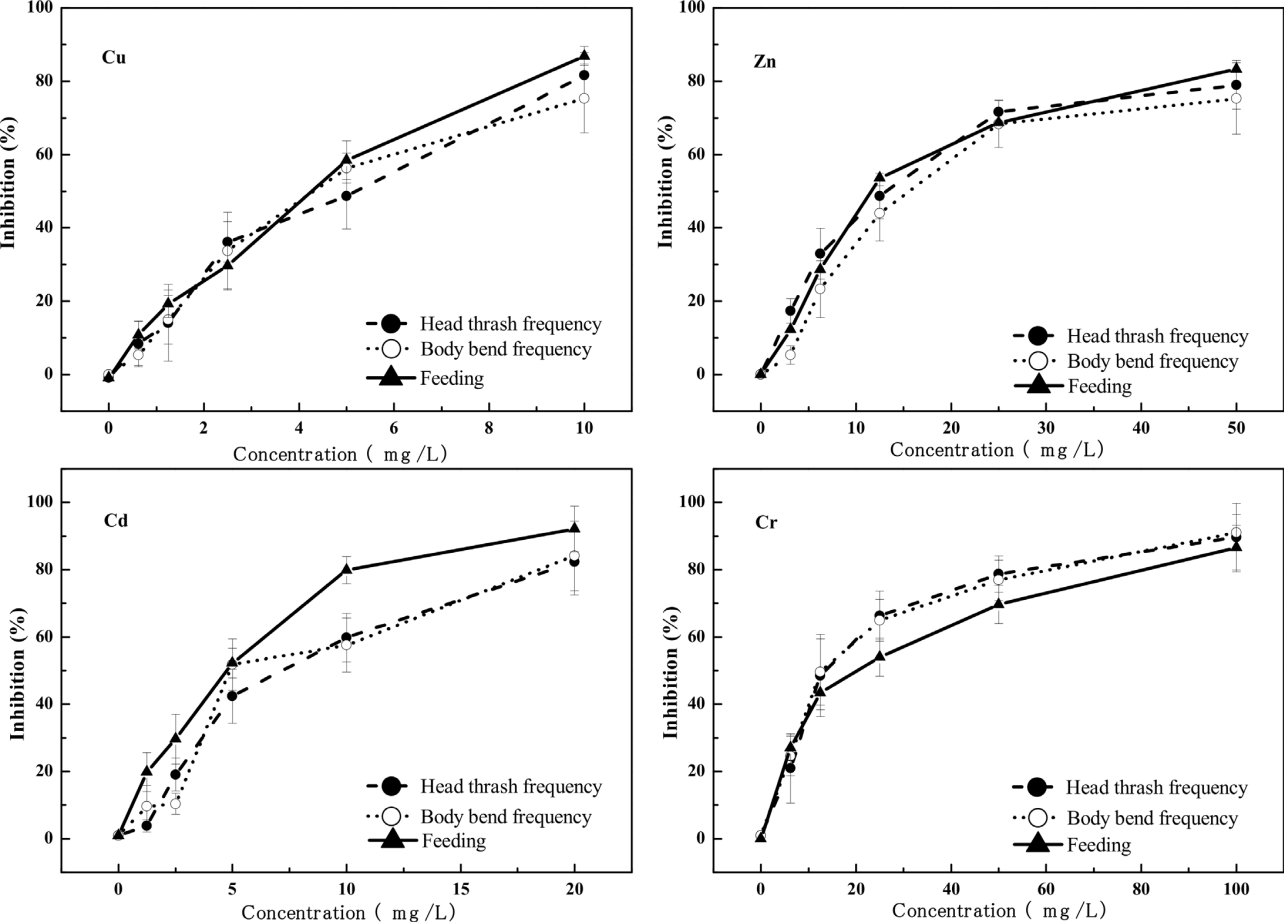
Concentration-response relationships for Cu, Zn, Cd, Cr exposures for head thrash frequency, body bend frequency, and feeding.

The concentration-response relationship of physiological and behavioral toxicity endpoints of the four heavy metals to *C*. *elegans* are shown in Figs 1 and 2. There is a good concentration-response relationship between the four heavy metals and the growth, reproduction, head thrash frequency, body bend frequency and feeding of *C*. *elegans*. The inhibition of each endpoint rose as the concentration of the heavy metals increased. The five physiological and behavioral endpoints were significantly (*p* <0.05) reduced for all test concentrations of Cu, Zn, Cd, and Cr, comparing with controls.

### 3.2 Enzymatic Activity Effect of Heavy Metals to *C*. *elegans*

**Fig 3:**
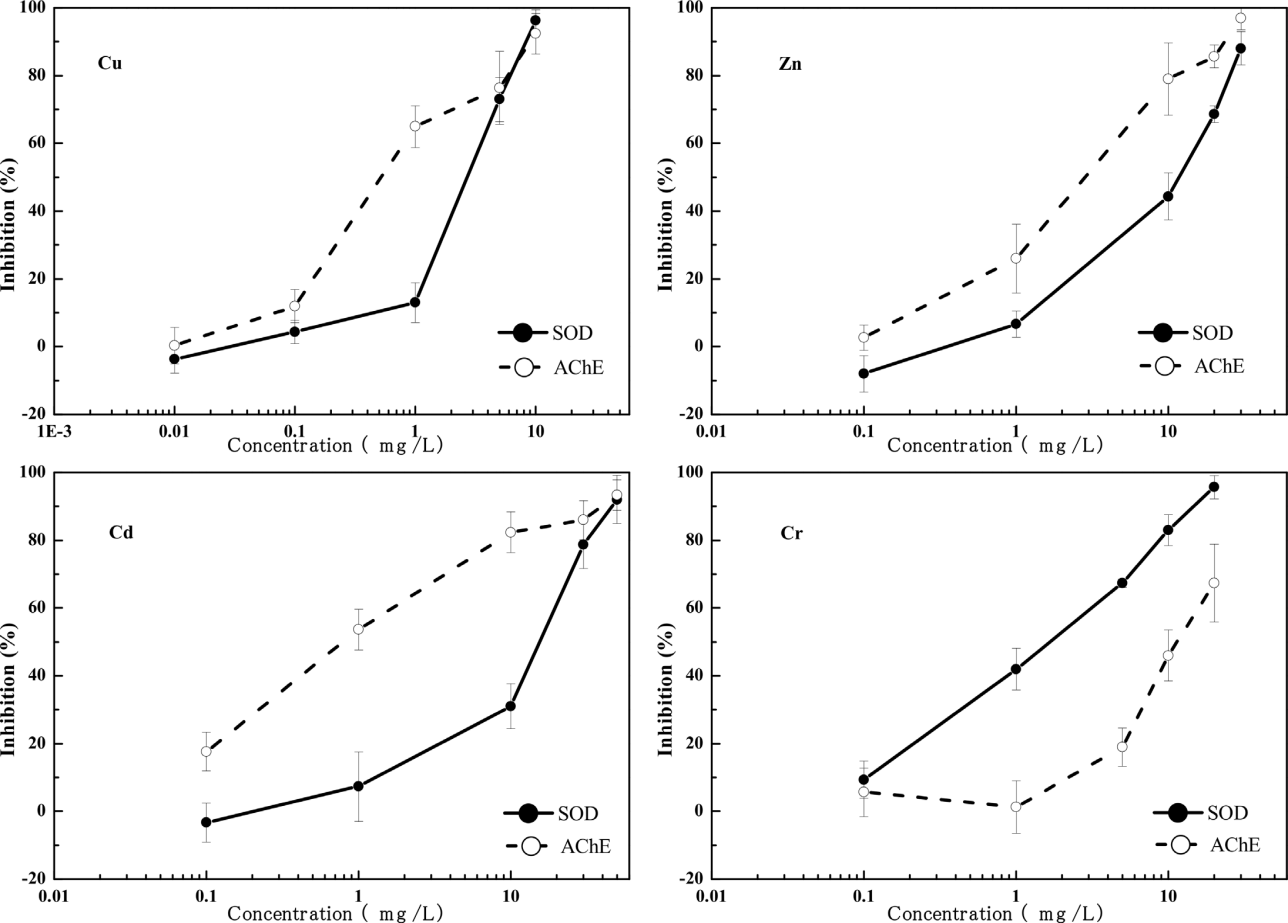
Concentration-response relationships for Cu, Zn, Cd and Cr exposures for acetylcholine esterase (AChE) and superoxide dismutase (SOD).

The concentration-response relationship of SOD and AChE toxicity endpoints of the four heavy metals to *C*. *elegans* are shown in Fig 3. There is a good concentration-response relationship between the heavy metals and the SOD and AChE activity inhibitions of *C*. *elegans*. The inhibition of each endpoint rose as the concentration of heavy metals increased. The two enzymatic endpoints were significantly (*p* <0.05) reduced from that of controls at all test concentrations of Cu, Zn, Cd, and Cr. The EC50 of AChE is significantly greater than that of SOD for Cr, indicating the sensitivity of SOD to Cr is greater than that of AChE; whereas the EC50 of SOD is significantly greater than that of AChE for Zn, Cd and Cu (*p* <0.05), indicating the sensitivity of AChE to Zn, Cd and Cu is greater than that of SOD.

The EC50 of AChE is significantly less than the EC50 of other physiological and behavioral endpoints for Cu, Zn, Cd to *C*. *elegans* (*p* <0.05), indicating that the sensitivity of AChE is the highest for Cu, Zn, and Cd. The EC50 of SOD for Cr to *C*. *elegans* is significantly less than the EC50 of other physiological and behavioral endpoints (*p* <0.05), indicating that the sensitivity of SOD is the highest for Cr.

## Discussion

For *C*. *elegans* few studies have provided direct comparison of the sensitivity of sublethal endpoints under common experimental conditions. The results of this study were very close to the findings reported previously [8, 9, 27, 28, 29, 30], and showed that there was a good concentration-response relationship between all four heavy metals and the sublethal toxicity effects to *C*. *elegans*.

Although the test methods of physiology and behavior of *C*. *elegans* are not identical, the results of this study, as well as previous findings, showed that the determination of physiological and behavioral toxicity endpoints of the heavy metals to *C*. *elegans* would produce similar results, even in different laboratories or by different researchers conducted, indicating a high measurement repeatability in these sublethal toxicity endpoints.

Superoxide dismutase (SOD) is the only enzyme in an organism that can directly use free radicals as substrate. It can catalyze disproportionation of the superoxide anion free radical (O^2-^) to generate H_2_O_2_ and O_2_, such that excessive free radicals in the cells are eliminated to avoid and decrease damages to the cells from oxyradicals [31]. Acetylcholine esterase (AChE) is an enzyme existing widely in the nerve system of animals, as well as a key enzyme in nerve conduction in organisms that plays an important role in the information conduction of the nerve system [32]. AChE is highly sensitive to organophosphorus pesticides, metals, detergents, etc., such that it has been widely applied in the detection of pollutants as a biomarker [33,34].

The experiment results indicate that heavy metals at low concentrations did not inhibit, but rather increased, the activities of SOD and AChE. This might attributable to the stress reaction to low-concentration heavy metal toxicants which would decrease the oxidative damage to the worms by heavy metals, thereby delaying aging and improving the lifespans of the nematodes. However, when exposed to high-concentration heavy metals, because the toxicity surpassed the detoxification capability of the *C*. *elegans*, the activities of these two enzymes were inhibited, thereby having observable effects to the growth, reproduction and behaviors of C. elegans at physiological levels [3,35–38].

However, the effects to the enzymatic activities of SOD and AChE vary among different heavy metals. At the same concentration, Cu, Zn and Cd can cause higher inhibition of AChE than that of SOD in the nematodes (except only that the inhibition of SOD is higher than that of SOD by Cu at 10 mg/L); whereas Cr, on the contrary, cause higher inhibition of SOD than that of AChE at the same concentration as other metals. This is mainly attributable to different toxicity mechanisms of the toxicants to the oxidoreductase system and nerve system of *C*. *elegans* [13,39,40].

Though lethality is the most common and most basic toxicity endpoint in toxicology, its insufficient sensitivity only enables it to reflect acute toxicity of materials, and it requires manual counting and artificial judgment of deaths for every sample, which affects the accuracy and increases errors in experimental determinations. Also, though growth and reproduction, as physiological endpoints, have quite high sensitivities, they share similar problems regarding determinations. Furthermore, the determination of reproduction requires a relatively long incubation period (72 hours) which is disadvantageous for quick tests. Other physiological endpoints, i.e. head thrash frequency and body bend frequency, have similar problems such as rather high degree of artificial interference and complicated, time-consuming determination process, thereby increasing instability in the data. On the other hand, the determination of feeding as a behavioral endpoint is relatively easier and, in contrast to traditional determination methods, the determination of absorbance in the solutions adopted automatic readings by using Microplate Reader in this experiment, which enabled simultaneous readings of 96 samples, thereby substantially decreasing operation time and room for artificial errors and increasing the accuracy and repeatability of the experiment. Moreover, SOD and AChE endpoints, although highly sensitive, consume enormous amounts of nematodes in experiments, requires much higher costs than all the toxicity endpoints mentioned above, and at the same time are easily subject to other influences causing substantial data fluctuations. Therefore, considering various aspects such as simplicity, accuracy, repeatability and costs of the experiments, feeding is the relatively ideal sublethal toxicity endpoint of heavy metals to the nematode *C*. *elegans*.

## Conclusion

There is a good concentration-response relationship between the heavy metals (Cu, Zn, Cd and Cr) and the sublethal toxicity effect to the nematode *Caenorhabditis elegans*, in terms of physiological, behavioral and enzymatic activity effects. The sensitivities of physiological and behavioral endpoints for four heavy metals are similar. AChE in *C*. *elegans* has the highest susceptibility to stress from Cu, Zn and Cd, while SOD has the highest susceptibility to stress from Cr. Through overall consideration of these sublethal endpoints, feeding is the relatively ideal sublethal toxicity endpoint of heavy metals to the nematode *C*. *elegans*, and therefore may provide technical support for time-efficient and accurate evaluation of ecological risks of heavy metals.
